# Case Report: Oral fecal microbiota transplantation in a Mediterranean spur-thighed tortoise (*Testudo graeca*) suffering from chronic gastrointestinal disease—procedure, clinical outcome and follow-up

**DOI:** 10.3389/fvets.2025.1560689

**Published:** 2025-05-21

**Authors:** Johannes Hetterich, Michael Pees

**Affiliations:** Department of Small Mammal, Reptile and Avian Medicine and Surgery, University of Veterinary Medicine Hannover, Foundation, Hannover, Germany

**Keywords:** reptile, tortoise, fecal microbiota transplantation, FMT, chronic gastrointestinal disease

## Abstract

**Introduction:**

Fecal microbiota transplantation (FMT) is the process of transferring fecal microbiota from a healthy donor into the gastrointestinal tract of a recipient. Although many mechanisms of FMT are still not completely understood at present, it has been described that the treatment of various gastrointestinal diseases in different species, including humans, is significantly improved by FMT therapy. Since the first report on FMT therapy in veterinary medicine in small mammals numerous cases have been reported, but little information has been published on the therapeutic effects of FMT treatment in reptiles. The present case report describes the effects of orally administered fecal microbiota transplantation in a Mediterranean spur-thighed tortoise (*Testudo graeca*) suffering from chronic gastrointestinal disorders.

**Case presentation:**

A nine-year-old, 330 g, intact female Mediterranean spur-thighed tortoise (*Testudo graeca*) from the animal owner’s own offspring was presented for consultation due to decreased general condition, anorexia and sialorrhea following oral intake of a lettuce species (*Lactuca virosa*) known for its poisonous plant ingredients (sesquiterpene lactones) 3 weeks prior to presentation. Pre-existing conditions were not reported. Clinical examination revealed sialorrhea and a reduced general condition. Diagnostic procedures included blood chemistry, radiography and ultrasonography. Despite repeated treatment attempts with various medical regimes over 158 days, the tortoise continued showing variable recurring gastrointestinal symptoms. An orally administered FMT was initiated and continued for a total of 3 weeks. Gastrointestinal signs improved rapidly within 1 week and resolved completely after 3 weeks. Over a follow up period of 9 months, no symptom recurrence or adverse effects were monitored.

**Conclusion:**

This case report describes the first successful trial of fecal microbiota transplantation in chelonians. The outcome indicates that this therapeutic approach may be beneficial not only to small animals but also for the therapy of gastrointestinal disorders in reptiles, especially those cases with insufficient conventional therapy results.

## Introduction

Fecal microbiota transplantation (FMT) is a treatment option that involves introducing fecal microbiota from a healthy donor into the recipient’s gastrointestinal tract (1). The treatment aims to positively influence the microbiome (and metabolom) and restore its balance (2, 3). The microbiota encompasses all microorganisms residing in the gastrointestinal tract (4, 5). These microorganisms have a crucial role in various physiological processes and form a symbiotic relationship with the host (6). Microbiome imbalances (dysbioses) are associated with various diseases, including acute and chronic gastrointestinal disorders (5). Although not all of the mechanisms of FMT are fully understood at present, it has been described that the treatment of various gastrointestinal diseases in different species, including humans, is significantly improved by FMT therapy (7–9). Anecdotally, FMT has been used as therapeutic approach in human medicine as early as the 4th century AD (10) and emerged as an important therapeutic tool in the 20th century, particularly for infections with *Clostridium difficile* (11). In veterinary medicine, the first case report of FMT treatment in a dog with Inflammatory Bowel Disease (IBD) was published in 2013 (12). Since then, numerous case reports and case series have been published for cats and dogs (13, 14), and also for other domestic animals such as horses (15). In the field of reptile medicine, a study evaluating the evolution of the microbiota in relation to behavioral aspects of green iguana hatchlings (*Iguana iguana*) was published as early as 1984 (16). In recent years, several studies have explored various aspects of microbiome research (17–19). The intestinal flora of wild and captive species of turtles has been described (20, 21). Another publication showed metagenomic differences in fecal microbiota between wild, released, and farmed individuals of a single turtle species (22). A recent study described host-microbial interactions in a desert lizard (*Eremias multiocellata*) and also assessed the biological impact of climate conditions on the gut microbiota (23). This study was also the first to perform fecal microbiota transplantation experiments in reptiles. However, no case reports or studies have examined the effects of FMT therapy in chelonians. The present report describes the treatment process, clinical outcome and follow-up of a tortoise with chronic gastrointestinal disease using FMT.

## Case presentation

### Clinical history

A nine-year-old, 330 g, intact female Mediterranean spur-thighed tortoise (*Testudo graeca*) bred from the owner’s own stock has been kept in a 25 m^2^ sized outdoor enclosure for the last 5 years alongside three siblings (one male, two females). Temperature, UVB-light and hibernation management were species-appropriate. The animals were fed daily, receiving hay (available ad libitum) and a variety of herbs and leafy green. The owner did not report any pre-existing conditions and the siblings were assessed as healthy. The tortoise was presented with a decreased general condition, anorexia and sialorrhea following oral intake of a lettuce species (*Lactuca virosa*) 3 weeks prior to presentation. Clinical symptoms developed within 1 day after oral ingestion and showed no improvement following soaking the tortoise repeatedly in warm water.

### Clinical findings and investigations

Clinical examination revealed a moderately softened plastron and minor chronic shell deformities. Sialorrhea and a moderately reduced general condition of the tortoise were also noted during the general examination. Initial diagnostic procedures included blood chemistry, radiography and ultrasonography. Blood chemistry examination was performed in an in-house laboratory (Cobas C 311, La Roche Ltd., Basel, Switzerland) 15 min after venipuncture (dorsal coccygeal vein) and revealed moderate hyperuricemia (6.5 mg/dL; reference range 0–5.2 mg/dL) (24). Radiographic examination (digital X-ray; detector system: Fujifilm Console Advance DR-ID 300 CL, Fujifilm Europe GmbH, Düsseldorf, Germany; tube system: Gierth X-ray International GmbH, Riesa, Germany; film focus distance 60 cm, 50 kV, 5 mA) under manual restraint in dorsoventral (vertical radiation beam direction) and lateral (horizontal radiation beam direction) projections showed a prominent filling of the digestive tract with ingesta. Ultrasonography (GE Vivid 7 Dimension, Micro curved array transducer, 5–9 MHz; GE Healthcare GmbH, Solingen, Germany) performed under manual restraint confirmed the sex diagnosis by clearly showing ovarian follicles but revealed no other abnormalities. Fecal samples were used for a microbiological examination including sensitivity testing conducted in a commercial veterinary laboratory at the time of starting antibiotic treatment. Testing revealed the growth of 2 gram-negative bacteria (*Cronobacter* spp. and *Citrobacter braakii*). Both bacteria showed intermediate susceptibility to enrofloxacine.

### Treatment and follow-up

**Figure 1:**
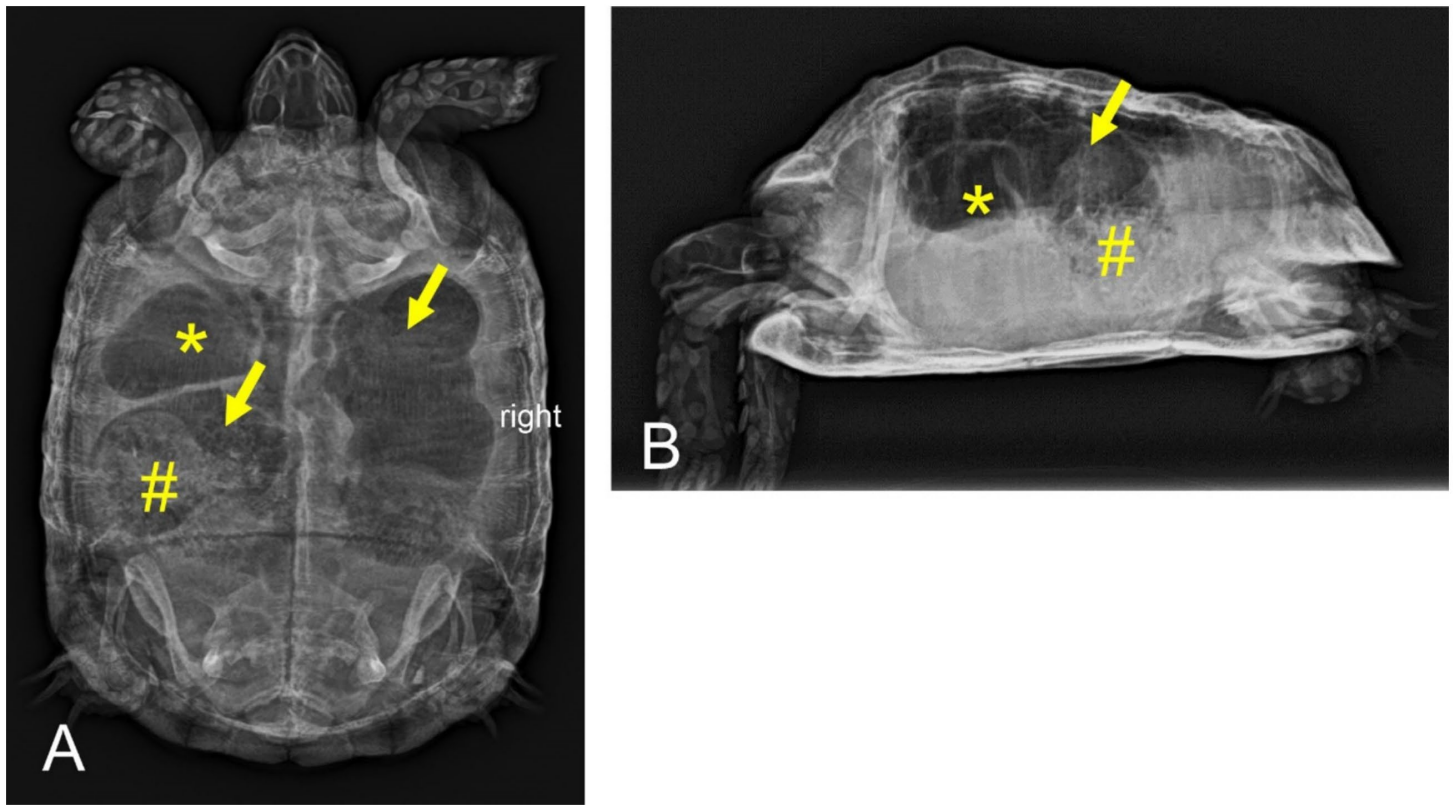
Dorsoventral **(A)** and horizontal-beam right lateral **(B)** radiographs of a nine-year-old Mediterranean spur-thighed tortoise (*Testudo graeca*) demonstrating severe gas accumulation throughout the gastrointestinal tract 33 days after initial presentation. The stomach (asterisk) and parts of the large intestine (arrows) appear to be filled only with gaseous contents. Parts of the large intestine, though, contain structured material (ingesta, number sign).

**Figure 2:**
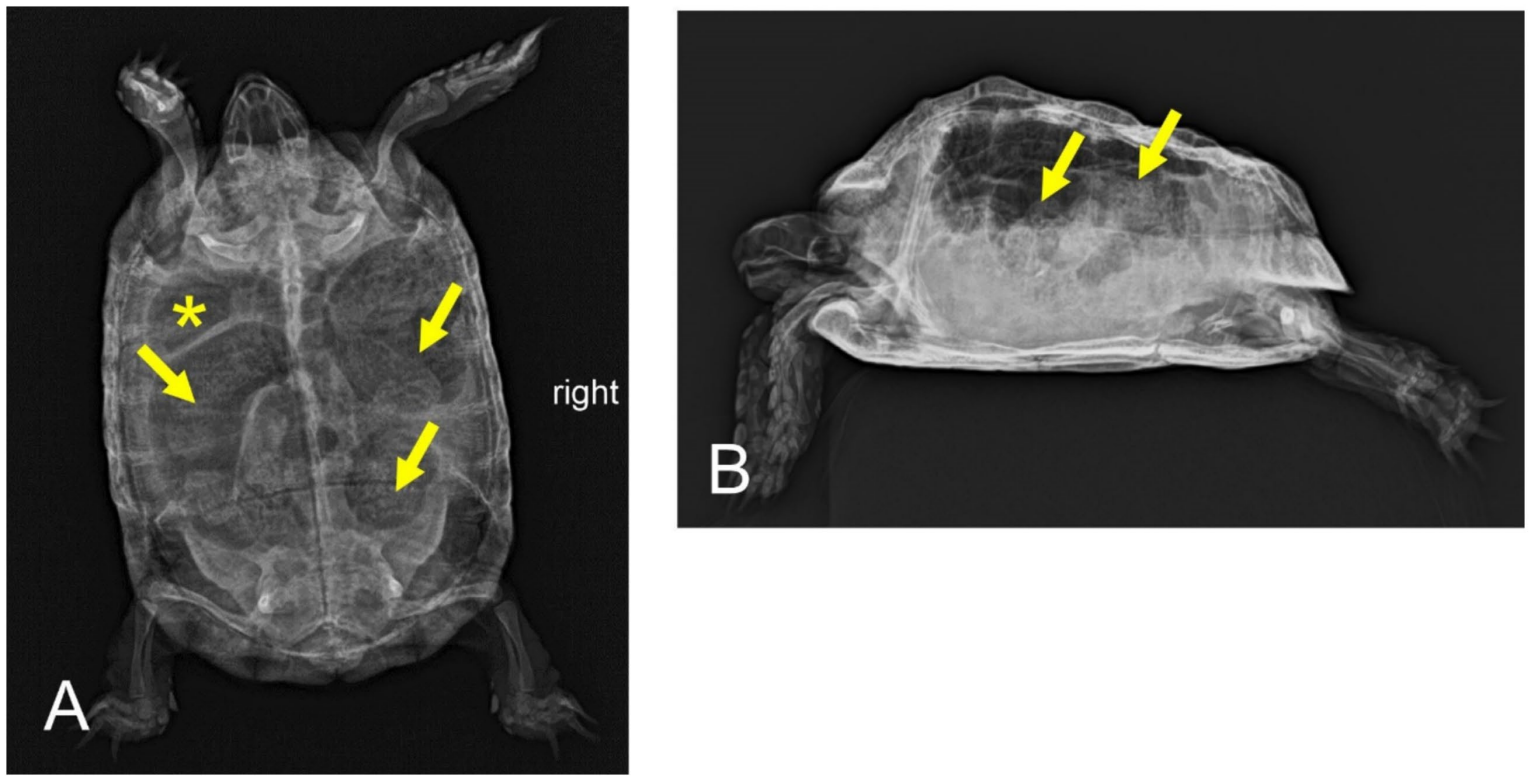
Dorsoventral **(A)** and horizontal-beam right lateral **(B)** radiographs of a nine-year-old Mediterranean spur-thighed tortoise (*Testudo graeca*) with persistent severe gas accumulation throughout the gastrointestinal tract 54 days after initial presentation. However, major parts of the gastrointestinal tract, including the stomach (asterisk) and various parts of the large intestine (arrows), clearly contain structured ingesta material.

**Figure 3:**
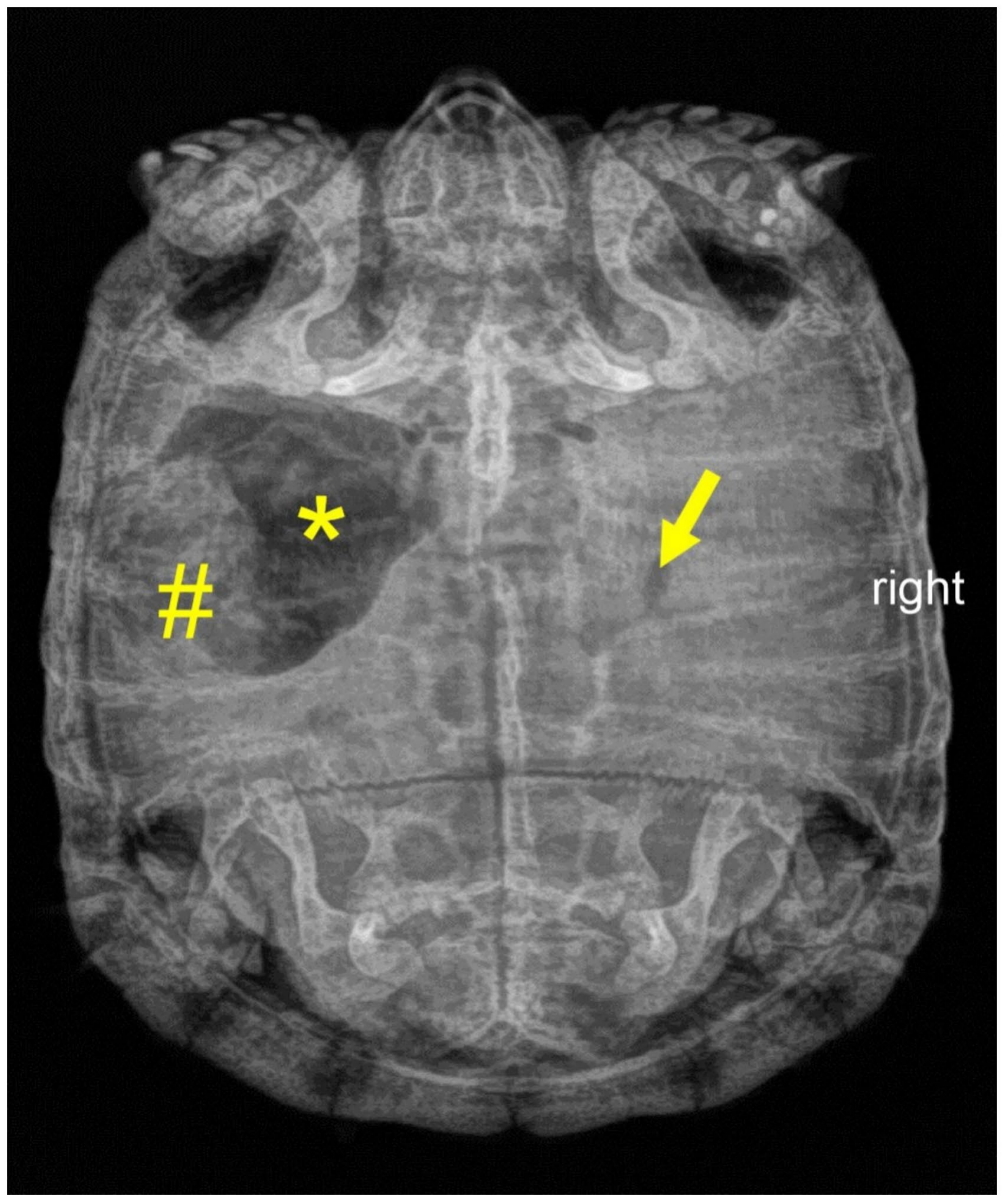
Dorsoventral radiograph of a nine-year-old Mediterranean spur-thighed tortoise (*Testudo graeca*) demonstrating mixed contents of gas (asterisk) and structured ingesta material (number sign) within the stomach 75 days after initial presentation. Note that only minor gas accumulation is still present in the intestine (arrow).

The animal received allopurinol (50 mg/kg SID PO; Allopurinol AL 100, Aliud Pharma, Laichingen, Germany) according to treatment recommendation (25) and parenteral fluids (10 mL/kg SID SC, Sterofundin, ISO 1/1 E; B. Braun AG, Melsungen, Germany). However, the tortoise’s general condition remained unchanged after 10 days of treatment and the animal was presented again. Neither a stationary therapy for 9 days nor several treatment approaches using various protocols including antibiotics, analgesics, probiotics, devolatilizing and gastroprotective drugs led to a sustainable and lasting therapy success in the following 149 days. Table 1 provides details on the respective gastrointestinal signs, diagnostic methods (including radiography of Figures 1–3), therapy attempts and outcomes of the follow-up history.

**Table 1:** Detailed information on the evolution of gastrointestinal signs and the corresponding diagnostic methods, therapies and outcomes of the according follow-ups during the entire case history.

Gastrointestinal signs	Diagnostics	Therapy	Outcome
Day 1			
Anorexia, sialorrhea, reduced overall condition and activity level	Radiography, sonography, blood chemistry	Parenteral fluids^1^, allopurinol^2^	Food intake returned; sialorrhea and reduced overall condition and activity level remained unaffected
Day 10 (+10)			
Sialorrhea, reduced overall condition and activity level	Blood hematology and SDMA-sampling, urine examination	Inhouse therapy for 9 days: parenteral fluids^1^, paramunity inducer^3^, forced feeding^4^, daily soaking in warm water	Apparent changes in feces quality parameters, sialorrhea and overall condition and activity level improved, no regular food intake
Day 33 (+23)			
Reduced food intake, postprandial sialorrhea, reduced overall condition, absent defecation, severe gastrointestinal tympanic distension	Radiography (Figure 1), microbiologic examination (feces)	Antibiotics (enrofloxacin^5^ and metronidazole^6^), tramadol^7^, simeticon^8^, probiotics^9^	Overall condition and activity level improved; regular defecation frequency, but reduced fecal quality; postprandial sialorrhea unaffected
Day 54 (+21)			
Postprandial sialorrhea, reduced overall condition and activity level, severe gastrointestinal tympanic distension	Radiography (Figure 2)	Antibiotics (enrofloxacin^5^ and metronidazole^6^), tramadol^7^, simeticon^8^, probiotics^9^	Food intake present but still decreased; undulating fecal quality; postprandial sialorrhea unaffected
Day 75 (+21)			
Slightly reduced overall condition and activity level, postprandial sialorrhea, painful defecation	Radiography (Figure 3)	Probiotics^9^	Regular food intake and fecal quality returned. Due to ongoing gastrointestinal signs, hibernation was not initiated
Day 131 (+56)			
Reduced overall condition and activity level, postprandial sialorrhea, reduced food intake, painful defecation	Radiography, esophagostomy and gastroscopy (in sedation)	Simeticon^8^, sucralfate^10^	All symptoms remained unaffected for the following 14 days
Day 145 (+14)			
Reduced overall condition and activity level, postprandial sialorrhea, reduced food intake, painful defecation	None	Bitter food compounds^11^, citric acid supplementation	Overall condition and activity level and food intake improved; postprandial sialorrhea and painful defecation remained unaffected
Day 159 (+14)			
Slightly reduced overall condition and food intake, postprandial sialorrhea, painful defecation	Radiography, blood chemistry and hematology examination, sonography	Start FMT treatment	Sialorrhea resolved and regular food intake was regained within 1 week. Painful defecation and fecal quality alterations resolved within 3 weeks. During this time period, the animal also regained a regular overall condition and activity level
Day 207 (+48)			
None	Blood chemistry, fecal examination (parasitologic)	None	Gastrointestinal disorders resolved during 3 weeks of FMT therapy. No adverse effects or symptom recurrence were observed.
Day 207–429			
None	None	None	The animal did not show any gastrointestinal signs during the further follow-up period of 222 days.

1 Allopurinol (50 mg/kg SID PO; Allopurinol AL 100, Aliud Pharma, Laichingen, Germany).

2 Parenteral fluids (10 mL/kg SID SC, Sterofundin, ISO 1/1 E; B. Braun AG, Melsungen, Germany).

3 Paramunity inducer (1 mL SC single injection; Zylexis®, Zoetis Deutschland GmbH, Berlin, Germany).

4 Feeding formula (15 mL/kg SID PO; Oxbow Critical Care Herbivore, Oxbow Pet Products, Murdock, NE, USA).

5 Enrofloxacin (10 mg/kg SID PO; Baytril 25 mg/mL, Elanco GmbH, Cuxhaven, Germany).

6 Metronidazole (30 mg/kg SID PO; Eradia 125 mg/mL, Virbac Tierarzeinmittel GmbH, Bad Oldesloe, Germany).

7 Tramadol (10 mg/kg every 48 h PO; Tramadolhydrochloride 50 mg/mL, Dechra Veterinary Products GmbH, Aulendorf, Germany).

8 Simeticon (1 mL/kg SID PO; Simeticon Dechra 41.2 mg/mL, Dechra Veterinary Products, Aulendorf, Germany).

9 Probiotics (0.2 g SID PO; Bene-Bac® gel, Dechra Veterinary Products, Aulendorf, Germany).

10 Sucralfate (50 mg/kg SID PO; Sucrabest®, Combustin GmbH, Hailtingen, Germany).

11 Bitter food compounds (chicory, dandelion).

#### FMT treatment

An orally administered fecal microbiota transplantation (FMT) was initiated as new therapeutic approach 181 days after the oral intake of lettuce and 159 days after the initial presentation. The feces were obtained from three clinically healthy siblings that had been housed and fed under identical conditions for the last 5 years. Fecal samples were macroscopically normal and tested negative for endoparasitic infections on two separate occasions prior to FMT. All four animals tested negative for herpesvirus (PCR and serology), reovirus (PCR) and mycoplasma (PCR) 3 years before. A total of 15 grams of fresh feces was mixed with 10 mL sodium chloride (sodium chloride 0.9%, B. Braun Melsungen AG, Melsungen, Germany) to a paste-like consistency. A total volume of 9 mL of dispersed fecal mixture was orally administered into the stomach in a two-step procedure (two-hour breaks between administrations) using a metal probe (diameter 6.4 mm, length 102 mm; Eickemeyer, Tuttlingen, Germany). The animal owner continued to administer fresh feces from the sibling animals through manual feeding every 2–3 days for a total period of 3 weeks. The fecal samples were mixed with sodium chloride and coated with the animal’s favorite salad. Using this procedure, voluntary oral ingestion of the transplants by the tortoise was achieved. Gastrointestinal signs resolved within 1 week (sialorrhea, reduced food intake) to 3 weeks (painful defecation) after the initial fecal transplantation. The tortoise’s defecations became more consolidated, displaying the physiological characteristics of normal feces. In addition, the animal regained a physiologic level of activity that had not been achieved during the entire former therapy period. Over the following 6 weeks, the animal did not exhibit any of the preceding gastrointestinal signs and continued food intake and defecation on a regular basis. No adverse effects were observed during this time period.

A final on-site follow-up 48 days after the initial fecal transplantation revealed a healthy overall condition. Regular food intake before and defecation with physiological fecal characteristics during the examination could be verified within the scope of the clinical follow-up. Blood chemistry parameters and parasitologic fecal sampling were normal. The tortoise was deemed to be free of any gastrointestinal signs, and the animal owner began preparations for a regular hibernation.

The animal was monitored for a total of 9 months following the initial FMT treatment. According to personal communication with the animal owner, the tortoise remained free of any gastrointestinal signs or adverse effects both before and after hibernation.

## Discussion

Gastrointestinal dysbiosis is considered a disruption of the normal physiological colonization of the gastrointestinal tract by microorganisms (5). FMT aims to correct an existing dysbiosis and consecutive metabolic imbalances (26). The detailed mechanisms of FMT in mammals are not yet fully understood, though various complex interactions between the host and its microbiota are assumed (3, 27, 28). The microbiota stimulates the development of the immune system and acts as a protective barrier against infectious agents (29), either through direct cell-to-cell contact mechanisms or through metabolites produced by the microbiota (30). Whenever there is a direct or indirect perturbation of the microbiota, the host becomes more susceptible to gastrointestinal infections (30). In human medicine, FMT treatment has been shown to have beneficial effects, not only for gastrointestinal disorders (31), but also for extragastrointestinal indications such as metabolic syndrome (32), autism, stereotypy, or speech disorders (33). FMT transplantation studies in small animals evaluated that fecal transplantation increased the number of bacterial diversity and particularly useful bacteria like, e.g., *Peptacetobacter hiranonis* and *Fusobacteria* ssp. (34, 35).

In addition, FMT treatment has been introduced to a number of wildlife species in recent years, bringing valuable therapy experience to more exotic, and possibly endangered species, for which novel treatment methods are needed (36–38). Despite the growing evidence supporting the application of FMTs to a wide range of animal species, there is a dearth of research on FMT, especially for carnivores (39).

In reptiles, a recent study described host-microbial interactions in a desert lizard (*Eremias multiocellata*) and also assessed the biological impacts of climate conditions on the gut microbiota (23). Interestingly, this study also included the first fecal microbiota transplantation experiments in reptiles showing that FMT enhanced antibacterial activity and host immune response of the lizards. This study provides useful initial data for future prospective evaluations of this emerging topic.

Detailed theoretical specifications have been published for the required characteristics of donor animals in small animal medicine (3, 26). In our case, three possible donor animals were available and considered to be suitable donors. The sibling tortoises were evaluated to be clinically healthy, showed regular food intake and defecation, and had no reported previous gastrointestinal disorders. Infections with intranuclear coccidiosis in tortoises (TINC) have been first described in radiated tortoises (*Astrochelys radiata*) (40). TINC can result in systemic disease, which has been described in several chelonian species (41), but is more common in tropical tortoises. However, TINC represents a chronic disease and there is a risk of transmission from asymptomatic carrier animals shedding coccidia for life (42). Therefore, sampling the siblings prior to FMT would have extended the donor assessment.

In general, FMT can be performed via the upper or lower gastrointestinal tract (43). In small animals, enemas, endoscopic transplantation into the intestine, and oral administration (capsule or dilution) have been described (9, 44–46). Nonetheless, no studies have evaluated any route of administration and their efficacy in reptiles. In the present case report, the authors chose oral administration, as tube-feeding with a metal probe is a common and low-risk procedure in the treatment of tortoises. In contrast, an incorrectly placed cloacal FMT may lead to a too rapid discharge from the rectum, which should be avoided.

FMT treatment protocols vary widely regarding the number and frequency of repeated administrations (26, 34, 47, 48). Chronicity of disease, route of administration and course of treatment represent important factors for the number of repeated FMT doses (26). The authors of this report aimed to establish a feasible treatment plan for continuing FMT therapy after the initial administration, considering defecation frequency of the siblings, suspected gastrointestinal transit time and animal habits (usual defecation time). Continuation of FMT may have been appropriate if the tortoise’s gastrointestinal signs had persisted.

Assessment of treatment efficacy should represent an important part of FMT evaluation. In several small animal studies, the short-term efficacy of FMT treatment was rated good to excellent (34, 35, 48–50). Often, medium-term efficacy remained unclear due to a lack of follow-up data. In our case, we considered a nine-month follow-up period after FMT initiation to be highly valuable to assess the long-term effect of the FMT procedure on the digestive system and the overall clinical outcome.

Studies evaluating the adverse effects of FMT have been published in human medicine (51) and, more recently, in small animal medicine (26, 52). Short- and medium-term adverse effects generally were rare and mostly mild and self-limiting (26, 48, 53). The FMT technique, transplant quality (54), potential comorbidities and immune competence of the recipient as well as the health of the donor were among the factors that needed to be considered for a safe FMT (26).

Gut microbial communities are often characteristic of specific dietary modes (55, 56). Obligate herbivory in mammals is associated with increased microbial diversity compared to other dietary modes (57). Herbivore reptiles, such as Galápagos tortoises and iguanids, share a similar digestive mode and gut morphology with mammalian hindgut fermenters (58). However, little is known about species-specific factors that influence the composition of gut microbiota in reptiles. A study evaluating gut microbial diversity in gopher tortoises (*Gopherus polyphemus*) found that fine-scale spatial structure, inbreeding, degree of relatedness and possibly ontogeny shape patterns of diversity in fecal microbiomes (59). Coprophagy may serve as another factor affecting the composition of the gut microbiome. Some species of herbivore reptiles have been described to exhibit coprophagy as normal behavior during microbiota development in juvenile life stages (16). Other species show regular coprophagy even in the presence of a good range of usual dietary components (60, 61). Although it is unknown, whether this behavior is the primary route of colonization for critical gut symbionts (59), evidence of coprophagy in wildlife may indicate that this behavior may be a physiological route to (re)establish a healthy microbiome, even in diseased individuals.

In general, it should be critically discussed whether the ingested lettuce plant actually caused the gastrointestinal symptoms. The temporal context and the available information on the toxicity of the plant for humans (62), other mammals (63) and reptiles (64) strengthen the assumption that the lettuce intake was the initial cause of the ongoing gastrointestinal disorders. *Lactuca virosa* is known for its poisonous plant ingredients (sesquiterpene lactones) (62, 64), which cause central nervous signs, but also gastrointestinal disorders in mammals (62, 63). However, no toxicological tests, such as spectrophotometric or chromatographic methods (65, 66), were carried out in this case to confirm the presence and amount of sesquiterpene lactones such as lactucerin, actucic acid, lactucopicrin and lactucin. Therefore, clear evidence of any association between the oral consumption of *Lactuca virosa* and gastrointestinal signs remains uncertain and speculative.

In the present report, husbandry parameters have been assessed as species-appropriate based on information from the animal owner, and all siblings were assessed without any abnormalities. However, moderate softening and deformities of the shell and uricemia are common signs of nephropathy (67, 68) and may also indicate possible nephropathy in this case. Intoxication with sesquiterpene lactones may have exacerbated renal disease, facilitating non-specific symptoms such as lethargy and reduced activity.

The conducted long-term gastrointestinal therapy prior to FMT treatment needs a critical reflection. The various treatment approaches using supportive therapy, including parenteral fluids, probiotics, gastroprotective therapy, antibiotics, or combinations of these treatments temporarily affected the various signs. Gastrointestinal tympanic distension and reduced food intake improved during antibiotic treatment. Antibiotics may have significantly altered the microbial composition and also reduced the amount of gas-forming gut bacteria, which can lead to tympanic distension. Probiotics may have positively altered the microbial composition and fecal quality. Also, by reducing the gastrointestinal pain of tympanic distension, the analgesics used may facilitated defecation frequency. Bitter food compounds also led to improved gastrointestinal signs, which may be related to the diverse immunomodulating, anti-inflammatory and digestive properties that bitter substances are known for (69, 70). However, other gastrointestinal signs, such as sialorrhea, remained largely unaffected, gastrointestinal signs recurred and no lasting improvement in clinical status was achieved. Subsequently, the conditions did improve rapidly after FMT initiation. However, recovery cannot be attributed solely to FMT, as the microbiome status and gastrointestinal signs were already influenced over a long period by the pre-treatments and their multiple (synergistic) effects as described above.

This case report also needs a critical review of antibiotic treatment regimes. In this report, antibiotic treatment was started using enrofloxacine and metronidazole when signs of gastrointestinal disease – suspected dysbiosis causing extended gastrointestinal tympania - were diagnosed. Fecal samples were sent to a laboratory for bacteriological examination and consecutive sensitivity testing. Especially the use of enrofloxacine is discussed critically for different reasons and is not recommended clearly as a first-line antibiotic in reptiles (71). However, in Germany enrofloxacine is approved for the treatment of gastrointestinal disease in reptiles, as the only antibiotic available. For this reason, the treatment regime was started and continued following an initial improvement of some gastrointestinal signs despite the laboratory results (intermediate sensitivity of enrofloxacine). Retrospectively, this measure needs to be viewed critically, as no lasting effect was seen and the use of both antibiotics in the present case may have had a significant (negative) impact on the tortoise’s microbiome. It is the authors’ experience and also reported in literature that antibiotic treatments of gastrointestinal disease in reptiles are often not effective (71). This case report is a more or less typical example of different common but unsuccessful treatment regimes. As treatment options for gastrointestinal disorders are not as versatile as in other companion animals, it was the authors’ aim to invent a new treatment option in this case, which could not be resolved using conventional therapies. Therefore, despite using antibiotics in the course of the treatment described here, this report is clearly intended to point out a new therapy alternative in FMT and minimize antibiotic use.

As it is inherent in case reports, several limitations arise due to individual circumstances. Most importantly, we are not able to associate the different clinical stages with the corresponding microbiome. Microbiota analysis would have been highly beneficial, especially comparing the gastrointestinal flora before and after FMT administration. Initial treatment was started before laboratory results were available, and treatment regimens were adapted individually to the clinical situation, without lasting effects. Although the animal was regularly presented to the clinic, some health assessments were conducted by the owner, and the interpretation of these could be subjective and potentially misleading. However, it is the nature of an individual case report that diagnostic and treatment procedures are case-sensitive and dependent on the owner’s compliance, which was excellent in this case. Finally, extrinsic or intrinsic factors other than FMT may have influenced clinical improvement. However, the tortoise’s rapid improvement after starting FMT, following several months of chronic, fluctuating gastrointestinal signs, suggests that FMT treatment likely had a positive impact on the therapy outcome.

Therefore, the scientific value of this report lies exclusively in the effective use of FMT as an alternative treatment option in a chronic case of gastrointestinal disease and pretreatment failures.

## Conclusion

This case report provides an evidence-based example of the use of FMT in a tortoise suffering from chronic gastrointestinal disease. Oral FMT proved to be a safe and non-invasive therapy approach. Following FMT therapy, chronic gastrointestinal symptoms vanished with no relapses over a nine-month follow-up period. Further studies are needed on the therapeutic efficacy of FMT in chelonians and all reptile orders.

## Data Availability

The original contributions presented in the study are included in the article/supplementary material, further inquiries can be directed to the corresponding author.

## References

[1] Borody TJ Khoruts A. Fecal microbiota transplantation and emerging applications. Nat Rev Gastroenterol Hepatol. (2012) 9:88–96. doi: 10.1038/nrgastro.2011.244, PMID: 22183182

[2] Cammarota G Ianiro G Tilg H Rajilić-Stojanović M Kump P Satokari R. European consensus conference on faecal microbiota transplantation in clinical practice. Gut. (2017) 66:569–80. doi: 10.1136/gutjnl-2016-313017, PMID: 28087657 PMC5529972

[3] Chaitman J Gaschen F. Fecal microbiota transplantation in dogs. Vet Clin North Am Small Anim Pract. (2021) 51:219–33. doi: 10.1016/j.cvsm.2020.09.012, PMID: 33131919

[4] Swanson KS Dowd SE Suchodolski JS Middelbos IS Vester BM Barry KA. Phylogenetic and gene-centric metagenomics of the canine intestinal microbiome reveals similarities with humans and mice. ISME J. (2011) 5:639–49. doi: 10.1038/ismej.2010.162, PMID: 20962874 PMC3105739

[5] Suchodolski JS. Analysis of the gut microbiome in dogs and cats. Vet Clin Pathol. (2022) 50:6–17. doi: 10.1111/vcp.13031, PMID: 34514619 PMC9292158

[6] Ziese AL Suchodolski JS. Impact of changes in gastrointestinal microbiota in canine and feline digestive diseases. Vet Clin North Am Small Anim Pract. (2021) 51:155–69. doi: 10.1016/j.cvsm.2020.09.004, PMID: 33131916

[7] Sugita K Yanuma N Ohno H Takahashi K Kawano K Morita H. Oral faecal microbiota transplantation for the treatment of *Clostridium difficile*-associated diarrhoea in a dog: a case report. BMC Vet Res. (2019) 15:11–4. doi: 10.1186/s12917-018-1754-z, PMID: 30616615 PMC6322325

[8] Borody TJ Eslick GD Clancy RL. Fecal microbiota transplantation as a new therapy: from Clostridioides difficile infection to inflammatory bowel disease, irritable bowel syndrome, and colon cancer. Curr Opin Pharmacol. (2019) 49:43–51. doi: 10.1016/j.coph.2019.04.017, PMID: 31173991

[9] Cerquetella M Marchegiani A Rossi G Trabalza-Marinucci M Passamonti F Isidori M. Case report: Oral fecal microbiota transplantation in a dog suffering from relapsing chronic diarrhea—clinical outcome and follow-up. Front Vet Sci. (2022) 9:893342. doi: 10.3389/fvets.2022.893342, PMID: 35859811 PMC9289623

[10] Zhang F Cui B He X Nie Y Wu K Fan D. Microbiota transplantation: concept, methodology and strategy for its modernization. Protein Cell. (2018) 9:462–73. doi: 10.1007/s13238-018-0541-8, PMID: 29691757 PMC5960466

[11] Eiseman B Silen W Bascom GS Kauvar AJ. Fecal enema as an adjunct in the treatment of pseudomembranous enterocolitis. Surgery. (1958) 44:854–9. PMID: 13592638

[12] Weese JS Costa MC Webb JA. Preliminary clinical and microbiome assessment of stool transplantation in the dog and cat. J Vet Intern Med. (2013) 3:705–5.

[13] Furmanski S Mor T. First case report of fecal microbiota transplantation in a cat in Israel. Isr J Vet Med. (2017) 72:35–41.

[14] Sugita K Shima A Takahashi K Ishihara G Kawano K Ohmori K. Pilot evaluation of a single oral fecal microbiota transplantation for canine atopic dermatitis. Sci Rep. (2023) 13:8824. doi: 10.1038/s41598-023-35565-y, PMID: 37258604 PMC10230478

[15] Mullen KR Yasuda K Divers TJ Weese JS. Equine faecal microbiota transplant: current knowledge, proposed guidelines and future directions. Equine Vet Educ. (2018) 30:151–60. doi: 10.1111/eve.12559, PMID: 32313396 PMC7159401

[16] Troyer K. Behavioral acquisition of the hindgut fermentation system by hatchling *Iguana iguana*. Behav Ecol Sociobiol. (1984) 14:189–93. doi: 10.1007/BF00299618, PMID: 40276788

[17] Abdelrhman KF Bacci G Mancusi C Mengoni A Serena F Ugolini A. A first insight into the gut microbiota of the sea turtle *Caretta caretta*. Front Microbiol. (2016) 7:1060. doi: 10.3389/fmicb.2016.01060, PMID: 27458451 PMC4935691

[18] Siddiqui R Maciver SK Khan NA. Gut microbiome–immune system interaction in reptiles. J Appl Microbiol. (2022) 132:2558–71. doi: 10.1111/jam.15438, PMID: 34984778

[19] Hoffbeck C Middleton DM Nelson NJ Taylor MW. 16S rRNA gene-based meta-analysis of the reptile gut microbiota reveals environmental effects, host influences and a limited core microbiota. Mol Ecol. (2023) 32:6044–58. doi: 10.1111/mec.17153, PMID: 37795930

[20] Fong JJ Sung YH Ding L. Comparative analysis of the fecal microbiota of wild and captive beal’s eyed turtle (*Sacalia bealei*) by 16S rRNA gene sequencing. Front Microbiol. (2020) 11:570890. doi: 10.3389/fmicb.2020.570890, PMID: 33240228 PMC7677423

[21] Niu X Lin L Zhang T An X Li Y Yu Y. Comparison of the intestinal flora of wild and artificial breeding green turtles (*Chelonia mydas*). Front Microbiol. (2024) 15:1412015. doi: 10.3389/fmicb.2024.1412015, PMID: 38873159 PMC11170157

[22] Khan I Bu R Ali Z Iqbal MS Shi H Ding L. Metagenomics analysis reveals the composition and functional differences of fecal microbiota in wild, farm, and released Chinese three-keeled pond turtles (*Mauremys reevesii*). Animals. (2024) 14:1750. doi: 10.3390/ani14121750, PMID: 38929370 PMC11201187

[23] Yang J Liu W Han X Hao X Yao Q Du W. Gut microbiota modulation enhances the immune capacity of lizards under climate warming. Microbiome. (2024) 12:37. doi: 10.1186/s40168-023-01736-2, PMID: 38388458 PMC10882899

[24] Andreani G Carpene E Cannavacciuolo A Di Girolamo N Ferlizza E Isani G. Reference values for hematology and plasma biochemistry variables, and protein electrophoresis of healthy Hermann’s tortoises (*Testudo hermanni* ssp.). Vet Clin Pathol. (2014) 43:573–83. doi: 10.1111/vcp.12203, PMID: 25285592

[25] Koelle P. Efficacy of allopurinol in European tortoises with hyperuricemia. Proc ARAV. (2001) 16:185–6.

[26] Winston JA Suchodolski J Gaschen FP Busch KB Marsilio S Costa MC. Clinical guidelines for fecal microbiota transplantation in companion animals. Adv Small Anim Care. (2024) 5:79–107. doi: 10.1016/j.yasa.2024.06.006 PMC13251538 42281763

[27] Kelly CR Kahn S Kashyap P Laine L Rubin D Atreja A. Update on fecal microbiota transplantation 2015: indications, methodologies, mechanisms, and outlook. Gastroenterology. (2015) 149:223–37. doi: 10.1053/j.gastro.2015.05.008, PMID: 25982290 PMC4755303

[28] Khoruts A Sadowsky MJ. Understanding the mechanisms of faecal microbiota transplantation. Nat Rev Gastroenterol Hepatol. (2016) 13:508–16. doi: 10.1038/nrgastro.2016.98, PMID: 27329806 PMC5909819

[29] Pilla R Suchodolski JS. The role of the canine gut microbiome and metabolome in health and gastrointestinal disease. Front Vet Sci. (2020) 6:498. doi: 10.3389/fvets.2019.00498, PMID: 31993446 PMC6971114

[30] Agus A Planchais J Sokol H. Gut microbiota regulation of tryptophan metabolism in health and disease. Cell Host Microbe. (2018) 23:716–24. doi: 10.1016/j.chom.2018.05.003, PMID: 29902437

[31] Khanna S Vazquez-Baeza Y González A Weiss S Schmidt B Muñiz-Pedrogo DA. Changes in microbial ecology after fecal microbiota transplantation for recurrent *C. difficile* infection affected by underlying inflammatory bowel disease. Microbiome. (2017) 5:1–8. doi: 10.1186/s40168-017-0269-3, PMID: 28506317 PMC5433077

[32] Zhang Z Mocanu V Cai C Dang J Slater L Deehan EC. Impact of fecal microbiota transplantation on obesity and metabolic syndrome—a systematic review. Nutrients. (2019) 11:2291. doi: 10.3390/nu11102291, PMID: 31557953 PMC6835402

[33] Vendrik KE Ooijevaar RE De Jong PR Laman JD Van Oosten BW Van Hilten JJ. Fecal microbiota transplantation in neurological disorders. Front Cell Infect Microbiol. (2020) 10:98. doi: 10.3389/fcimb.2020.00098, PMID: 32266160 PMC7105733

[34] Chaitman J Guard B Sarwar F Lidbury J Steiner J Suchodolski J. Fecal microbial transplantation decreases the dysbiosis index in dogs presenting with chronic diarrhea. J Vet Intern Med. (2017) 31:1287.

[35] Niina A Kibe R Suzuki R Yuchi Y Teshima T Matsumoto H. Fecal microbiota transplantation as a new treatment for canine inflammatory bowel disease. Biosci Microbiota Food Health. (2021) 40:98–104. doi: 10.12938/bmfh.2020-049, PMID: 33996366 PMC8099633

[36] Thacher PR Kendrick EL Maslanka M Muletz-Wolz CR Bornbusch SL. Fecal microbiota transplants modulate the gut microbiome of a two-toed sloth (*Choloepus didactylus*). Zoo Biol. (2023) 42:453–8. doi: 10.1002/zoo.21751, PMID: 36629092

[37] Bornbusch SL Harris RL Grebe NM Roche K Dimac-Stohl K Drea CM. Antibiotics and fecal transfaunation differentially affect microbiota recovery, associations, and antibiotic resistance in lemur guts. Anim Microbiome. (2021) 3:65. doi: 10.1186/s42523-021-00126-z, PMID: 34598739 PMC8485508

[38] Kohl KD Weiss RB Cox J Dale C Dearing MD. Gut microbes of mammalian herbivores facilitate intake of plant toxins. Ecol Lett. (2014) 17:1238–46. doi: 10.1111/ele.12329, PMID: 25040855

[39] Bornbusch SL Crosier A Gentry L Delaski KM Maslanka M Muletz-Wolz CR. Fecal microbiota transplants facilitate post-antibiotic recovery of gut microbiota in cheetahs (*Acinonyx jubatus*). Commun Biol. (2024) 7:1689. doi: 10.1038/s42003-024-07361-5, PMID: 39715825 PMC11666765

[40] Jacobse ER Schuhmacher J Telford SR Greiner EC Buergelt CD Gardiner CH. Intranuclear coccidiosis in radiated tortoises (*Geochelone radiata*). J Zoo Wildl Med. (1994) 25:95–102.

[41] Kolesnik E Dietz J Heckers KO Marschang RE. Detection of intranuclear coccidiosis in tortoises in Europe and China. J Zoo Wildl Med. (2017) 48:328–34. doi: 10.1638/2016-0054R1.1, PMID: 28749291

[42] Wellehan JF Jacobsen E Stilwell J Gibbons PM Garner MM Rosenoff E. Testudine Intranuclear coccidiosis (TINC). J Herpetol. (2022) 32:144–54. doi: 10.5818/JHMS-D-20-00024

[43] Kelly BJ Tebas P. Clinical practice and infrastructure review of fecal microbiota transplantation for *Clostridium difficile* infection. Chest. (2018) 153:266–77. doi: 10.1016/j.chest.2017.09.002, PMID: 28923757 PMC5812771

[44] Schmitz SS. Observational study of small animal practitioners’ awareness, clinical practice and experience with fecal microbiota transplantation in dogs. Top Companion Anim Med. (2022) 47:100630. doi: 10.1016/j.tcam.2022.100630, PMID: 35021112

[45] Gal A Barko PC Biggs PJ Gedye KR Midwinter AC Williams DA. One dog’s waste is another dog’s wealth: a pilot study of fecal microbiota transplantation in dogs with acute hemorrhagic diarrhea syndrome. PLoS One. (2021) 16:e0250344. doi: 10.1371/journal.pone.0250344, PMID: 33872339 PMC8055013

[46] Berlanda M Innocente G Simionati B Di Camillo B Facchin S Giron MC. Faecal microbiome transplantation as a solution to chronic enteropathies in dogs: a case study of beneficial microbial evolution. Animals. (2021) 11:1433. doi: 10.3390/ani11051433, PMID: 34067662 PMC8156139

[47] Pereira GQ Gomes LA Santos IS Alfieri AF Weese JS Costa MC. Fecal microbiota transplantation in puppies with canine parvovirus infection. J Vet Intern Med. (2018) 32:707–11. doi: 10.1111/jvim.15072, PMID: 29460302 PMC5867004

[48] Toresson L Spillmann T Pilla R Ludvigsson U Hellgren J Olmedal G. Clinical effects of faecal microbiota transplantation as adjunctive therapy in dogs with chronic enteropathies—a retrospective case series of 41 dogs. Vet Sci. (2023) 10:271. doi: 10.3390/vetsci10040271, PMID: 37104426 PMC10145442

[49] Bottero E Benvenuti E Ruggiero P. Fecal microbiota transplantation (FMT) in 16 dogs with idiopatic IBD. Veterinaria. (2017) 31:31–45. doi: 10.5555/20173098113

[50] Niina A Kibe R Suzuki R Yuchi Y Teshima T Matsumoto H. Improvement in clinical symptoms and fecal microbiome after fecal microbiota transplantation in a dog with inflammatory bowel disease. Vet Med. (2019) 10:197–201. doi: 10.2147/VMRR.S230862, PMID: 31819862 PMC6898721

[51] Michailidis L Currier AC Le M Flomenhoft DR. Adverse events of fecal microbiota transplantation: a meta-analysis of high-quality studies. Ann Gastroenterol. (2021) 34:802–14. doi: 10.20524/aog.2021.0655, PMID: 34815646 PMC8596209

[52] Lee MA Questa M Wanakumjorn P Kol A McLaughlin B Weimer BC. Safety profile and effects on the peripheral immune response of fecal microbiota transplantation in clinically healthy dogs. J Vet Intern Med. (2024) 38:1425–36. doi: 10.1111/jvim.17061, PMID: 38613431 PMC11099722

[53] Rojas CA Entrolezo Z Jarett JK Jospin G Kingsbury DD Martin A. Microbiome responses to fecal microbiota transplantation in cats with chronic digestive issues. Vet Sci. (2023) 10:561. doi: 10.3390/vetsci10090561, PMID: 37756083 PMC10537086

[54] Takáčová M Bomba A Tóthová C Micháľová A Turňa H. Any future for faecal microbiota transplantation as a novel strategy for gut microbiota modulation in human and veterinary medicine? Life. (2022) 12:723. doi: 10.3390/life12050723, PMID: 35629390 PMC9146664

[55] Muegge BD Kuczynski J Knights D Clemente JC Gonzalez A Fontana L. Diet drives convergence in gut microbiome functions across mammalian phylogeny and within humans. Science. (2011) 332:970–4. doi: 10.1126/science.1198719, PMID: 21596990 PMC3303602

[56] Delsuc F Metcalf JL Wegener Parfrey L Song SJ González A Knight R. Convergence of gut microbiomes in myrmecophagous mammals. Mol Ecol. (2014) 23:1301–17. doi: 10.1111/mec.12501, PMID: 24118574

[57] Ley RE Hamady M Lozupone C Turnbaugh PJ Ramey RR Bircher JS. Evolution of mammals and their gut microbes. Science. (2008) 320:1647–51. doi: 10.1126/science.1155725, PMID: 18497261 PMC2649005

[58] Bjorndal KA. Fermentation in reptiles and amphibians In: Mackie RI White BA, editors. Gastrointestinal Microbiology. Boston, MA: Springer (1997). 199–230.

[59] Yuan ML Dean SH Longo AV Rothermel BB Tuberville TD Zamudio KR. Kinship, inbreeding and fine-scale spatial structure influence gut microbiota in a hindgut-fermenting tortoise. Mol Ecol. (2015) 24:2521–36. doi: 10.1111/mec.13169 25809385

[60] Lance VA Morafka DJ. Post natal lecithotroph: a new age class in the ontogeny of reptiles. Herpetol Monogr. (2001) 15:124–34. doi: 10.2307/1467040, PMID: 39964225

[61] Joshua QI Hofmeyr MD Henen BT. Seasonal and site variation in angulate tortoise diet and activity. J Herpetol. (2010) 44:124–34. doi: 10.1670/08-306R1.1

[62] Besharat S Besharat M Jabbari A. Wild lettuce (*Lactuca virosa*) toxicity. BMJ Case Rep. (2009) 2009:bcr0620080134. doi: 10.1136/bcr.06.2008.0134, PMID: 21686920 PMC3031874

[63] Nozohour Y Jalilzadeh-Amin G Maham M. First case report of toxicity with *Lactuca virosa* in a lamb. Fut Nat Prod. (2021) 6:56–62.

[64] Schramm R. LandschildkrötenFutterpflanzen: das Bestimmungsbuch im Taschenformat / Ricarda Schramm. Grebenhain: Tartaruga-Verlag (2017).

[65] Salapovic H Geier J Reznicek G. Quantification of sesquiterpene lactones in Asteraceae plant extracts: evaluation of their allergenic potential. Sci Pharm. (2013) 81:807–18. doi: 10.3797/scipharm.1306-17, PMID: 24106675 PMC3791941

[66] Merfort I. Review of the analytical techniques for sesquiterpenes and sesquiterpene lactones. J Chromatogr A. (2002) 967:115–30. doi: 10.1016/S0021-9673(01)01560-6, PMID: 12219925

[67] Selleri P Hernandez-Divers SJ. Renal diseases of reptiles. Vet Clin North Am Exot Anim Pract. (2006) 9:161–74. doi: 10.1016/j.cvex.2005.10.008 16407084

[68] Holz P. Diseases of the urinary tract In: Doneley B Monks D Robert Johnson BC, editors. Reptile medicine and surgery in clinical practice. Hoboken, NJ: John Wiley & Sons (2017). 323–30.

[69] Rezaie P Bitarafan V Horowitz M Feinle-Bisset C. Effects of bitter substances on GI function, energy intake and glycaemia-do preclinical findings translate to outcomes in humans? Nutrients. (2021) 13:1317. doi: 10.3390/nu13041317, PMID: 33923589 PMC8072924

[70] Qiao K Zhao M Huang Y Liang L Zhang Y. Bitter perception and effects of foods rich in bitter compounds on human health: a comprehensive review. Food Secur. (2024) 13:3747. doi: 10.3390/foods13233747, PMID: 39682819 PMC11640738

[71] Weese JS. Antimicrobial therapy in reptiles In: Dowling PM Prescott JF Baptiste KE, editors. Antimicrobial therapy in veterinary medicine (2024). 767–89.

